# Purification and Evaluation of the Biological Activity of Recombinant Mouse Prolactin

**DOI:** 10.4014/jmb.2503.03029

**Published:** 2025-07-14

**Authors:** Yoon-A Shin, Holim Jin, Yongjin Lee, Han-Byeol Shin, Young-Jin Son

**Affiliations:** 1 Department of Pharmacy, Sunchon National University, Suncheon 57922, Republic of Korea; 2 Department of Nutritional Science & Food Management, Ewha Womans University, Seoul 03760, Republic of Korea; 3 Abrain Inc., Research and Development Division, Suwon 16328, Republic of Korea

**Keywords:** Prolactin, MBP, affinity chromatography, purification, *E. coli*, 3T3-L1

## Abstract

Prolactin, a hormone secreted by lactotroph cells in the anterior pituitary lobe, plays a crucial role in vascular growth and immune homeostasis. In this study, we evaluated the functional activity of recombinant mouse prolactin produced using *Escherichia coli*. By employing a maltose-binding protein tag and TEV protease cleavage, we enhanced protein solubility and stability while ensuring high bioactivity. The recombinant prolactin was purified to over 99% purity through a streamlined two-step chromatography process, as confirmed by SDS-PAGE and Western blot analyses. Cell-based assays verified that the purified prolactin retained its biological activity, indirectly demonstrating its structural and functional stability. Additionally, the cost-effectiveness and simplicity of this method were highlighted by the reusability of the affinity column, reducing purification costs compared to conventional methods. This study provides a simple purification method for obtaining activated recombinant mouse prolactin, suggesting its potential use in biological and biochemical research.

## Introduction

Prolactin, a polypeptide hormone consisting of 199 amino acids and with a molecular weight of approximately 23 kDa, is produced and released by lactotroph cells located in the anterior pituitary gland. Structurally analogous to growth hormones, prolactin undergoes various post-translational modifications, including phosphorylation and sulfation, which contribute to its structural diversity and functional versatility [1, 2]. The predominant form of prolactin in circulation is a 23 kDa monomer, but it also appears in high-molecular-weight complexes known as big prolactin and macroprolactin [3]. These complexes are created by the binding of monomeric prolactin to IgG autoantibodies [4]. Although these larger forms have minimal biological activity and no confirmed pathological role, emerging research suggests they may modulate immune responses in certain conditions [5, 6]. Additionally, prolactin can be cleaved by proteases into smaller variants, including forms of 14, 16, and 22 kDa. These variants demonstrate prolactin’s adaptability and may serve specialized roles, further illustrating the complexity of prolactin’s structural and functional landscape [3, 5].

Prolactin is involved in a broad spectrum of physiological processes. Notably, it is essential in angiogenesis, where it promotes blood vessel growth, a critical function during pregnancy that supports fetal development. Prolactin regulates immune responses by modulating the functionality of immune cells, including lymphocytes and macrophages, thereby contributing to the preservation of immunological homeostasis [1, 3, 7]. This is associated with autoimmune diseases and suggests that it may play a role in the regulation of inflammation [7, 8]. Osmoregulation is another important function of prolactin, as it helps regulate electrolyte and water balance within the body. This ability to manage fluid balance underscores prolactin’s role in maintaining homeostasis, particularly in response to stress and environmental changes [9]. In reproductive biology, prolactin is critical for maintaining the menstrual cycle, facilitating pregnancy, and enabling milk production postpartum [10]. Its regulation of the reproductive system emphasizes its relevance in both fertility and maternal health. Because of its broad physiological roles, prolactin levels are frequently measured in clinical diagnostics, particularly for conditions such as prolactinomas (prolactin-secreting tumors), menstrual irregularities, infertility, and other reproductive disorders [11, 12]. Given its diverse roles, prolactin serves as an important biomarker for assessing endocrine health, making precise measurement essential for clinical and research purposes [12].

Both mouse and human adipose tissues express Prolactin receptor and its expression notably increases during the adipogenesis of bone marrow stromal cells [13-19]. This suggests that Prolactin may affect not only breast tissue but also energy homeostasis, adipogenesis, and lipid metabolism. Previous studies have reported that Prolactin increases PPARγ expression while inhibiting FAS expression in 3T3-L1 preadipocytes [20, 21]. In addition, one study found that Prolactin inhibits adipogenesis in adipose tissue by reducing the activities of LPL, acetyl-CoA carboxylase, and fatty acid synthase (FAS) during the lactation period in mice [22]. Pituitary adenomas that secrete excessive Prolactin may be associated with obesity, and some patients with prolactinomas or obesity have been reported to experience weight loss when serum Prolactin levels are normalized [23, 24]. Taken together, these findings strongly suggest that Prolactin plays a crucial role in regulating adipocyte function.

*Escherichia coli* is widely employed as a host organism for recombinant protein production because of its high efficiency in protein expression, cost-effectiveness, and adaptability to both laboratory research and industrial-scale applications [25]. Recombinant prolactin expressed in *E. coli* has facilitated studies on prolactin’s physiological and pharmacological functions, supporting applications in diagnostics and therapeutics [26, 27]. This capability highlights the importance of efficient expression systems in the large-scale production of high-purity proteins needed for research and potential clinical applications. However, despite these advantages, high-level expression in *E. coli* can lead to protein misfolding and the accumulation of insoluble aggregates [28]. To solve this problems, fusion tags such as MBP have been used. MBP enhances solubility and facilitates affinity-based purification. It is also known to increase the expression levels of eukaryotic proteins in *E. coli*, making it a widely used tool in recombinant protein expression systems [29, 30].

Recombinant mouse prolactin has previously been expressed in *E. coli* for biochemical and functional studies. Previous studies on the purification of recombinant mouse prolactin reported its expression in the form of inclusion bodies, requiring denaturation and refolding to restore biological activity, thereby making the complex process and labor-intensive [31]. In another study, prolactin was expressed in the periplasm and purified through multi-step processes involving RP-HPLC and HPSEC. However, this approach is complex and costly [32]. These examples demonstrate the feasibility of an *E. coli*-based system for prolactin production but also highlight the limitations of traditional purification approaches. Typically, recombinant protein purification involves complex procedures, multiple chromatography steps, low yields, or the risk of contamination [33, 34]. Although the use of affinity tags and protease-based cleavage systems has improved efficiency, technical challenges remain, including incomplete tag removal and scalability issues [35]. Therefore, simplified and high-purity purification strategies are needed to make prolactin more widely and practically applicable in biological and medical studies.

This study aims to obtain biologically active recombinant mouse prolactin (mPRL) using *E. coli*, employing a simple and cost-effective two-step chromatography approach with a maltose-binding protein (MBP) tag. The proposed method enhances protein solubility and stability, ensuring high bioactivity while maintaining a streamlined purification process.

## Materials and Methods

### Cloning of Recombinant Mouse Prolactin Gene in Bacterial Expression Vector

DNA fragments encoding recombinant mouse prolactin were cloned into the bacterial expression vector pMAL-c2X. The recombinant mouse prolactin sequence was designed based on reference data from GenBank (NIH, USA) and synthesized as pBHA-mPRL (Bioneer, Republic of Korea). The codon usage of the synthetic sequence was optimized for *E. coli* expression, and its codon adaptation index (CAI) was calculated to be 0.89 using an *E. coli* K-12 codon usage table, indicating that the sequence is well optimized for bacterial expression. To ensure directional cloning, restriction enzyme sites for *Nde*I (NEB, USA) and *Xho*I (NEB, USA) were incorporated. To prevent cleavage of the MBP coding sequence, a modified pMAL-c2X vector without *Nde*I cleavage site in the MBP gene was used. A preliminary experiment comparing *E. coli* DH5α and BL21 revealed similar expression levels of the recombinant prolactin, indicating that DH5α was suitable as the expression host under the given experimental conditions. The vector system used in this study also enabled effective IPTG-induced expression in this host, allowing successful production of soluble recombinant protein.

### Expression of Recombinant Mouse Prolactin in Flask Culture

*E. coli* DH5α cells transformed with recombinant plasmids encoding MBP-fused mPRL were grown in 5 ml of LB medium containing 100 μg/ml ampicillin (Sigma-Aldrich, USA) at 37°C to serve as an inoculum for the main culture. When the optical density at 600 nm (OD_600_) reached 0.5–0.7, the seed culture was transferred to 500 ml of LB medium for the main culture. After 4 h of growth at 37°C, when the OD_600_ reached 0.5–0.7, 1 mM IPTG (Bioneer) was added, and the temperature was lowered to 25°C to induce protein expression for 23 h. The temperature reduction to 25°C facilitates proper protein folding and enhances the solubility of MBP-fused mPRL by mitigating aggregation and misfolding, which are more prevalent at higher expression temperatures.

### Cell Lysis and Supernatant Collection

Cells were harvested by centrifugation at 8,000 rpm for 10 min at 4°C. The pellet was resuspended in 25 ml of buffer A (50 mM Tris, 0.5 mM EDTA, 5% glycerol, pH 8.0) per gram of cell weight. Cells were then disrupted via sonication (Sonics & Materials Inc., USA) using 30% amplitude with 3-sec pulses and 5-sec intervals for a total of 15 min on ice. The lysate was centrifuged at 8,000 rpm for 10 min at 4°C, and the supernatant containing the soluble MBP-fused recombinant mouse prolactin was collected and filtered through a 0.45 μm membrane. Protein concentrations were measured using the Bradford assay with bovine serum albumin (BSA) as the standard according to a general manual.

### Primary Purification of Recombinant Mouse Prolactin by MBP Affinity Chromatography

The filtered supernatant was loaded onto a 5 ml MBP Trap HP column (GE Healthcare Life Sciences, USA) pre-equilibrated with 72 ml of buffer A. Following a wash with 36 ml of buffer A, elution was performed using 72 ml of buffer B (50 mM Tris, 0.5 mM EDTA, 5% glycerol, 20 mM maltose monohydrate, pH 8.0). The eluted fractions containing MBP-fused recombinant mouse prolactin were analyzed by SDS-PAGE using a 12% gel (Bio-Rad, USA) and stained with Coomassie Brilliant Blue (Sigma-Aldrich). Stained protein bands were quantified using the ImageJ program (http://imagej.nih.gov/ij), and fractions containing the target protein were pooled.

### Cleavage of MBP-Fusion Tag by TEV Protease

To remove residual maltose, buffer exchange and concentration were performed using an Amicon ultracentrifuge filter with a 10-kDa molecular weight cutoff (Merck, USA). The MBP-fused prolactin protein purified via the MBP affinity column underwent TEV protease treatment (1 mg TEV per 5 mg protein) to facilitate the cleavage of the MBP fusion tag. The TEV enzyme was purified and supplied in our laboratory. In order to express recombinant TEV (rTEV) protease, we used TEV expression cells containing of pET-TEV vector. After cell culture with LB medium, we purified rTEV protease with His-tag chromatography [36]. purified rTEV protease was added in an amount of 1.92 mg to 8 ml sample solution with a concentration of 1.2 mg/ml. The reaction mixture was incubated at 20°C for 16 hours, and the cleavage of recombinant prolactin was confirmed by SDS-PAGE analysis performed using a 12% gel, followed by Coomassie Brilliant Blue staining. The protein bands were quantified with the ImageJ program.

### Secondary Purification of Recombinant Mouse Prolactin with MBP Affinity Chromatography

The ultrafiltrated sample was re-applied to the MBP affinity column, pre-equilibrated with 20 ml of buffer A. Following sample loading, the column was washed with 10 ml of buffer A and eluted using 20 ml of buffer B. Fractions containing recombinant mouse prolactin were analyzed by SDS-PAGE, stained with Coomassie Brilliant Blue, and quantified using ImageJ. The stained protein bands were pooled based on the analysis. Subsequently, buffer change and enrichment were performed with DW.

### Western Blot Analysis of Recombinant Mouse Prolactin

The identity and purity of the final purified recombinant mouse prolactin were confirmed by western blot analysis. An extracted protein was resolved through 10% SDS-PAGE and subsequently transferred onto a polyvinylidene difluoride (PVDF) membrane (Amersham Biosciences, USA). The membranes were incubated with primary antibodies against mPRL (Thermo Fisher Scientific, USA) and β-actin (Cell Signaling Technology, USA) at 4°C for 16 h. Subsequently, the membranes were reacted with an HRP-conjugated secondary antibody, Goat anti-Rat IgG H&L (Abcam, UK) and Goat anti-mouse IgG H&L (Abcam), at room temperature for 2 h. After washing TBS containing 1% Tween 20, the membrane was developed using the SuperSignal West Pico Chemiluminescent Substrate (Pierce Chemical, USA), and protein band intensity was analyzed using iBright CL1500 Imaging System (Thermo Fisher Scientific).

### Cell Culture

3T3-L1 preadipocytes were obtained from the Korean Cell Line Bank and cultured in Dulbecco's Modified Eagle's Medium (DMEM; Welgene, Republic of Korea) supplemented with 10% BCS and 100 U/ml penicillin/streptomycin. The cells were maintained in an incubator at 37°C with 5% CO_2_.

### Cytotoxicity Analysis of Recombinant Mouse Prolactin

To determine the effect of recombinant mouse prolactin on 3T3-L1 cells, the cells were seeded at 2 × 10^4^ cells per well in a 96-well cell culture plate. The next day, 100 ng/ml of recombinant mouse prolactin in 10% FBS DMEM was added, and the cells were cultured for three days. Then, cell viability was analyzed using the Cell Counting Kit-8 (CCK-8) (Dojindo Molecular Technologies, Japan).

Recombinant Mouse Prolactin Confirms the Expression of mRNA Genes Rrelated to Adipocyte Differentiation To determine the effect of recombinant mouse prolactin on the mRNA expression of genes related to adipocyte differentiation, 3T3-L1 cells were seeded at 6 × 10^5^ cells per well in a 6-well cell culture plate. The next day, the cells were treated with 100 ng/ml of recombinant mouse prolactin in a differentiation-inducing medium (MDI; 0.5 μM 3-isobutyl-1-methylxanthine (IBMX), 1 μM dexamethasone (DEX), and 10 μg/ml insulin) and cultured for 3 days. After that, the medium was replaced with a maturation-promoting medium (10% FBS DMEM + 10 μg/ml insulin) supplemented with 100 ng/ml of recombinant mouse prolactin, and the cells were cultured for a total of 6 days, with the medium being replaced every 2 days. Following the culture period, mRNA was extracted using TRIzol (Invitrogen, USA), and cDNA was synthesized using the M-MLV cDNA synthesis kit (Enzynomics). PCR was then performed, with glyceraldehyde-3-phosphate dehydrogenase (GAPDH) used as an internal control for gene expression analysis. The base sequences of the genes used in the experiment are shown in Table 2.

### Statistical Analysis

The results of this study were repeated three times, and statistical differences were analyzed using Student’s *t*-tests. A probability value (p-value) of less than 0.05 was considered statistically significant (p-value: *p < 0.05, **p < 0.01, ***p < 0.001).

## Results

### Construction of the Vector for Recombinant Mouse Prolactin

**Fig. 1:**
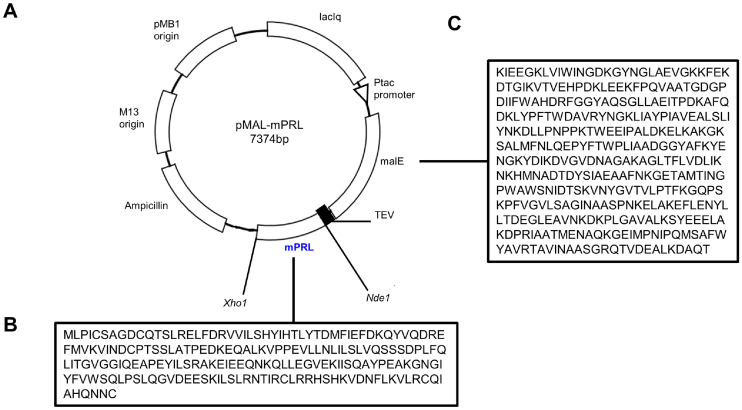
Construction of the recombinant mouse prolactin expression vector. (**A**) Recombinant mouse prolactin expression vector map. (**B**) Amino acid sequence of recombinant mouse prolactin without signal peptide. (**C**) Amino acid sequence of MBP.

The pMAL-mPRL construct was engineered to express MBP-fusion mPRL utilizing a modified pMAL-c2X vector, which incorporates a TEV protease cleavage site for subsequent separation of the MBP tag (Fig. 1). The Prolactin insert and pMAL vector were subjected to restriction enzyme digestion with *Nde*I and *Xho*I, followed by ligation and transformation into *E. coli* DH5α cells. Successful plasmid construction was confirmed through colony PCR and restriction enzyme digestion, verifying the presence of the recombinant pMAL-mPRL plasmid.

### Expression of MBP-Fusion Recombinant Mouse Prolactin in Cell Culture

**Fig. 2:**
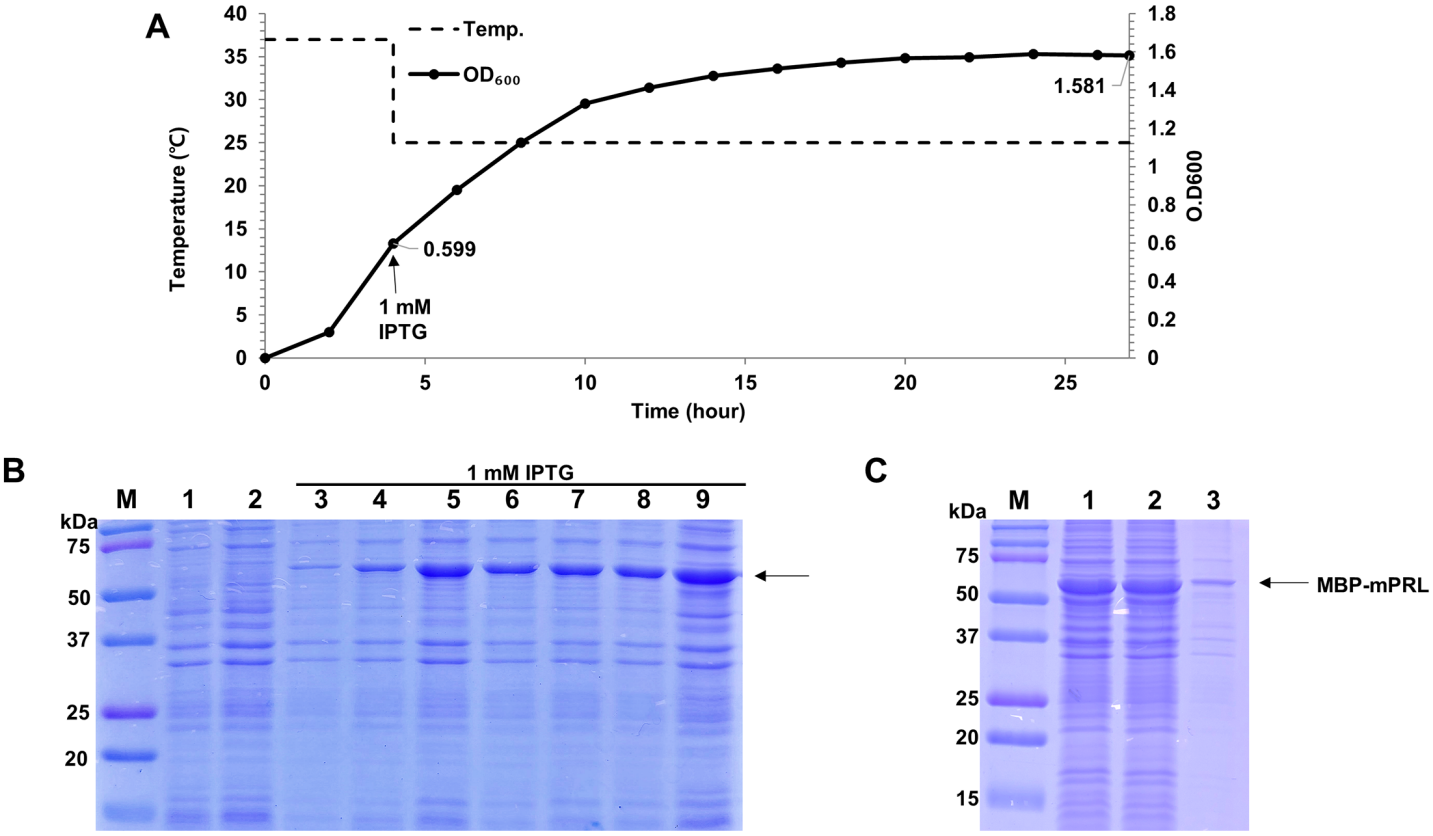
Expression patterns of according to culture and induction time of recombinant mouse prolactin expressing cells using flask. (**A**) Cell growth curve of recombinant mouse prolactin. Cells were cultured for 4 h at 37°C and induced with 1 mM IPTG for 23 h at 25°C (**B**) SDS-PAGE analysis of the expressed recombinant mouse prolactin. M: protein size marker; Lane 1-2: before induction; Lane 3-9: after induction for 1 h, 3 h, 6 h, 10 h, 14 h, 18 h, and 23h; The arrow indicates MBP fusion recombinant mouse prolactin. (**C**) SDS-PAGE analysis of the supernatant and pellet. M: protein size marker; Lane 1: Cell lysate; Lane 2: pellet of cell lysate; Lane 3: supernatant of cell lysate. The arrow indicates MBP fusion recombinant mouse prolactin.

Following induction, MBP-fusion mPRL-expressing cells were cultured at 25°C. To monitor the expression kinetics, samples were collected at specific time intervals post-induction, with SDS-PAGE analysis revealing a substantial increase in MBP-fusion mPRL expression over time (Fig. 2B). Peak expression was observed after 20 h, after which expression levels began to decline (Fig. 2A). As determined spectrophotometrically at the time of peak expression, the optical density of the culture at OD_600_ reached 1.58, and its expression level was quantified to 19.88% using the ImageJ assay (Fig. 2B, lane 9). The molecular weight of the expressed MBP-fusion mPRL was approximately 60.5 kDa.[Table T1]

### Purification of Recombinant Mouse Prolactin via MBP Affinity Chromatography

**Fig. 3:**
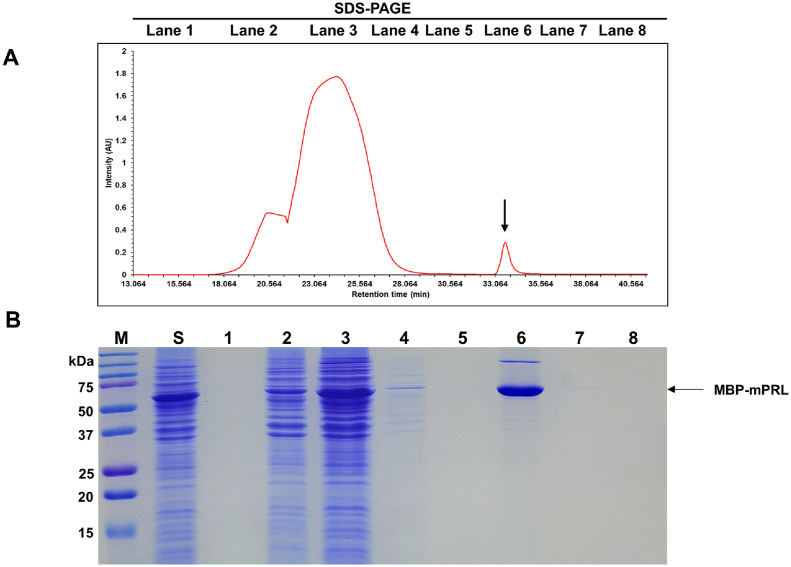
First affinity chromatogram of recombinant mouse prolactin using MBP column. (**A**) Wavelength was measured at 280 nm. The arrow indicates MBP fusion recombinant mouse prolactin. (**B**) SDS-PAGE analysis of recombinant mouse prolactin after first affinity chromatography. M: protein size marker; S: Sample; Lane 1: Fraction during sample loading step; Lane 2-4: Fraction during washing step; Lane 5-8: Fraction during elution step; The arrow indicates MBP fusion recombinant mouse prolactin.

After conducting centrifugation to collect cells, a wet pellet weighing 2.45 g was obtained from the 500 ml fermentation medium. As shown in Fig. 2C, the expressed MBP-fusion mPRL was produced in a soluble form and was found in the supernatant following cell lysis. The total protein concentration of the MBP-fusion mPRL solution was 207.84 mg. The collected supernatant was loaded onto an MBP affinity column equilibrated with buffer A. The MBP affinity column was then washed with buffer A and eluted with buffer B. According to the SDS-PAGE analysis, the purified MBP-fusion mPRL eluted with buffer B, and most impurities were removed during the loading and washing steps (Fig. 3B, lane 6).

**Fig. 4:**
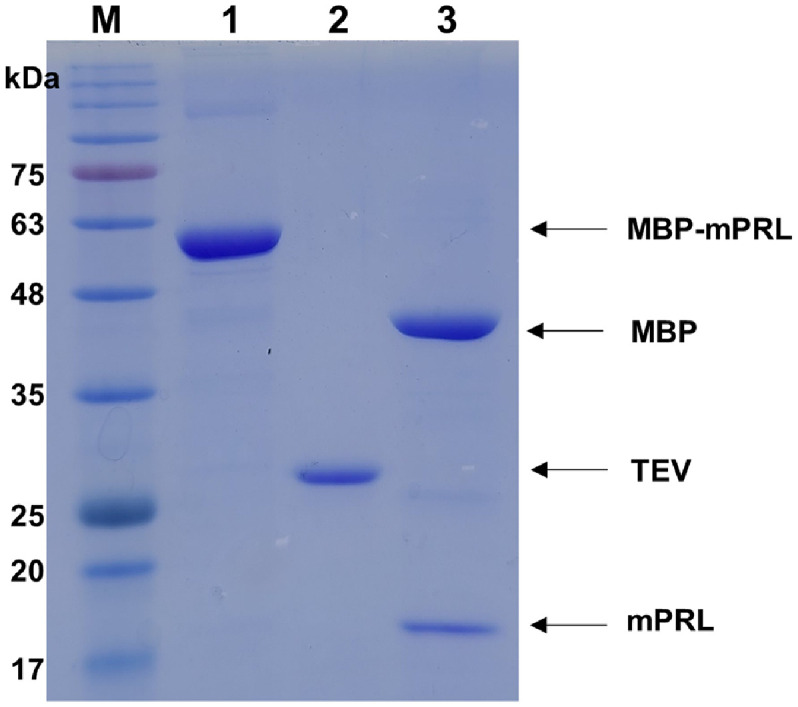
SDS-PAGE analysis of MBP fusion recombinant mouse prolactin cleavage using recombinant TEV protease. M: protein size marker; Lane 1: MBP fusion recombinant mouse prolactin after the first purification; Lane 2: TEV protease; Lane 3: cleaved product of the MBP fusion recombinant mouse prolactin by TEV protease.

Maltose was removed from the solution using buffer A, followed by cleavage of the fusion protein. The removal of maltose was essential, as the MBP affinity column could not be reused without this step. To separate mPRL from MBP, TEV protease was added to the MBP-fusion mPRL solution eluted from the MBP affinity column. To optimize cleavage conditions, different temperatures (4°C, 20°C, and 30°C) and TEV-to-protein ratios were tested, and the best conditions were selected. Incubation at 20°C overnight with a 1:5 ratio ensured stability of both the fusion protein and TEV protease while achieving efficient cleavage. TEV protease cleaved at the TEV recognition site between mPRL and MBP, and SDS-PAGE analysis confirmed complete cleavage (Fig. 4, lane 3, and Fig. 6A, lane 2). After TEV protease cleavage, mPRL (18 kDa) and MBP (42.5 kDa) appeared on the SDS-PAGE gel. Additionally, the TEV protease band was visible on the SDS-PAGE gel. Following the initial MBP affinity column and TEV protease cleavage, 9.45 mg of protein was obtained.

**Fig. 5:**
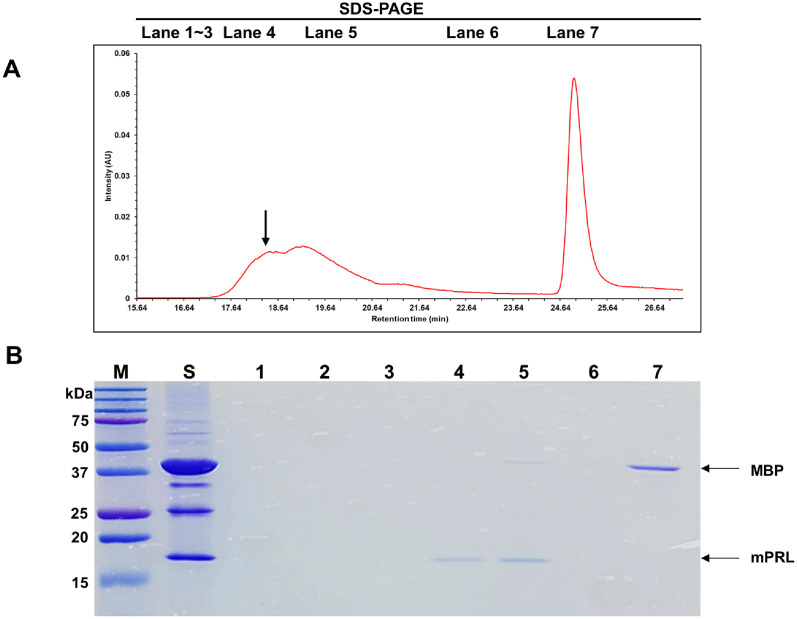
Second affinity chromatogram of recombinant mouse prolactin using MBP column. (**A**) Wavelength was measured at 280 nm. The arrow indicates recombinant mouse prolactin (**B**) SDS-PAGE analysis of recombinant mouse prolactin after second affinity chromatography. M: protein size marker; S: Sample; Lane 1: Fraction during sample loading step; Lane 2-5: Fraction during washing step; Lane 6-7: Fraction during elution step.

**Fig. 6:**
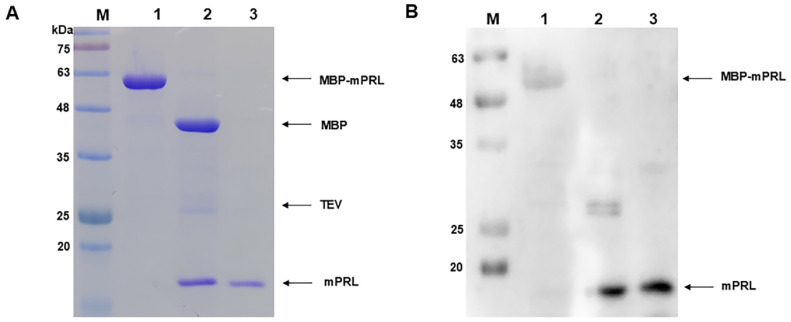
Analysis of the purified recombinant mouse prolactin. (**A**) SDS-PAGE analysis of the purified recombinant mouse prolactin. M: protein size marker; Lane 1: MBP fusion recombinant mouse prolactin after the first purification; Lane 2: Cleaved product of the MBP fusion recombinant mouse prolactin by TEV protease; Lane 3: The final purified recombinant mouse prolactin after the purification process. (**B**) Western blot analysis of the purified recombinant mouse prolactin. M: protein size marker; Lane 1: MBP fusion recombinant mouse prolactin after the first purification; Lane 2: Cleaved product of the MBP fusion recombinant mouse prolactin by TEV protease; Lane 3: The final purified recombinant mouse prolactin after the purification process.

When the solution containing cleaved mPRL and MBP was loaded onto the MBP affinity column, the cleaved mPRL did not bind to the column's functional groups, whereas the cleaved MBP did. The fractions that passed through the column during the loading and washing steps contained purified mPRL, while the fractions eluted with buffer B contained cleaved MBP due to the presence of maltose in the buffer (Fig. 5B, lane 4). The cleaved mPRL was separated from MBP by exploiting MBP’s binding affinity for the chromatographic resin in the MBP affinity column. Then buffer changed to DW and concentrated. The purification process is summarized in Table 2. Ultimately, the final yield of purified mPRL from the 500 ml fermentation medium was 0.57 mg, with its expression level quantified as 99.66% using ImageJ analysis (Fig. 6A, lane 3). The final purified mPRL was validated by western blot analysis, which confirmed its expression and detected a signal corresponding to the expected molecular weight of the purified protein (Fig. 6B, lane 3).

### Evaluation of the Cellular Activity of Recombinant Mouse Prolactin

**Fig. 7:**
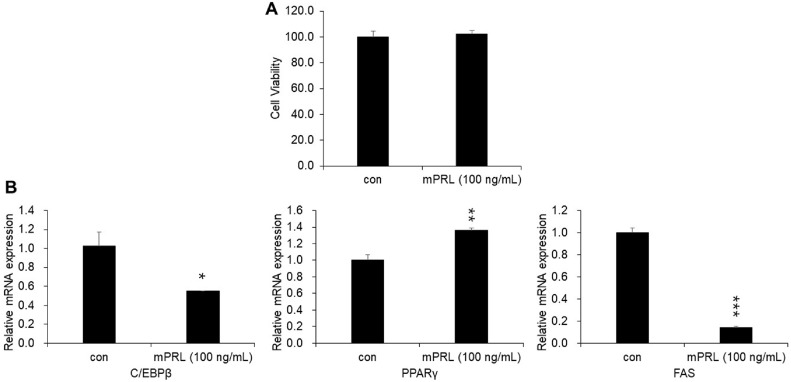
Effect of recombinant mouse prolactin on the mRNA expression of genes associated with adipocyte differentiation. (**A**) CCK-8 assay was conducted to evaluate the cytotoxicity of recombinant mouse prolactin in 3T3-L1 cells. (**B**) After treating 3T3-L1 cells with recombinant mouse prolactin, changes in the mRNA expression of C/EBPβ, PPARγ, and FAS, which are genes related to adipocyte differentiation, were confirmed by real-time qPCR. Data are presented as mean ± SEM from three independent replicates. Statistical significance is indicated as follows: * *P* < 0.05, ** *P* < 0.01, *** *P* < 0.001 (*n* = 3, Student’s *t*-test).

Since previous research has reported that prolactin affects the mRNA expression of genes related to adipocyte differentiation, this study treated 3T3-L1 cells with purified recombinant mouse prolactin to assess its cytotoxicity and its effects on the mRNA expression of adipocyte differentiation-related genes. As a result, no cytotoxicity was observed following treatment with purified recombinant mouse prolactin (Fig. 7A). Additionally, after treatment, the mRNA expression levels of C/EBPβ and FAS, which are involved in adipocyte differentiation, decreased, whereas the mRNA expression of PPARγ increased (Fig. 7B). These findings are consistent with previous studies, confirming the biological activity of purified recombinant mouse prolactin in this study [20-22].

## Discussion

This study presents a streamlined and efficient method for the production and purification of mPRL using a bacterial expression system. The pMAL-c2X expression vector was chosen for this study due to its design optimized for efficient recombinant protein production. It features an MBP tag to enhance solubility and simplify purification processes. Additionally, the vector includes a lac operator for IPTG-inducible expression control, allowing precise regulation of protein production [37]. By leveraging a fusion protein strategy incorporating an MBP tag and TEV protease cleavage, the protocol achieved high-purity mPRL with a simplified process. The results demonstrate that the MBP fusion tag effectively enhanced the solubility and stability of the recombinant protein, consistent with previous findings highlighting its role in mitigating aggregation and degradation of target proteins [38, 39]. Additionally, the use of TEV protease enabled precise cleavage of the MBP tag, preserving the structural integrity of the purified mPRL [40].

The two-step purification process, which included an initial MBP affinity chromatography followed by a re-purification step after cleavage, proved to be highly effective. The dialysis step to remove residual maltose from buffer B was particularly crucial in maintaining column reusability, highlighting the cost-efficiency of the protocol. In the second purification step, when the solution of TEV-cleaved mPRL and MBP was loaded onto the MBP affinity column, the cleaved mPRL did not bind to the column's functional groups and was effectively collected in the flow-through during the washing step. Meanwhile, the presence of maltose in buffer B facilitated the selective elution of cleaved MBP during the elution step. This approach ensured the efficient separation of mPRL from MBP and highlights the utility of this method in achieving high-purity protein while minimizing contamination. Such approaches align with cost-saving strategies in affinity chromatography and have been successfully implemented in scalable purification processes [41].

The final purified mPRL exhibited a purity of over 99%, as confirmed by SDS-PAGE and Western blot analyses. SDS-PAGE showed that the recombinant mPRL appeared at approximately 18 kDa due to the removal of the signal peptide, while Western blot verified its expression and presence in the purified sample, supporting the reliability of the purification process.

3T3-L1 cells are a preadipocyte lineage that differentiate into adipocytes when appropriately stimulated [42]. Insulin, insulin-like growth factor-1, epidermal growth factor, and platelet-derived growth factor are known to promote adipogenesis in 3T3-L1 cells, with fetal bovine serum (FBS) acting as a particularly potent stimulant [43, 44]. In an early lipid metabolism study, prolactin was reported to increase lipoprotein lipase (LPL) activity and triglyceride storage in 3T3-L1 cells, raising the possibility that prolactin might substitute FBS in inducing 3T3-L1 cell differentiation [45, 46]. Additionally, while prolactin dose-dependently increased C/EBPβ mRNA expression in NIH-3T3 cells, this effect was not observed in 3T3-L1 cells. However, prolactin increased PPARγ mRNA expression in both NIH-3T3 and 3T3-L1 cells when combined with adipogenic hormones such as MIX and DEX [20]. On the other hand, another study reported that prolactin reduces the protein and mRNA expression of fatty acid synthase (FAS) in 3T3-L1 adipocytes [21]. These findings suggest that Prolactin may have a dual function in regulating adipocyte gene expression. Therefore, in this study, purified recombinant prolactin was administered to 3T3-L1 cells to measure the mRNA expression levels of C/EBPβ, PPARγ, and FAS-genes associated with adipocyte differentiation—and to indirectly evaluate the biological activity of prolactin by comparing the results with previous studies. As a result, the mRNA expression of C/EBPβ and FAS decreased, whereas PPARγ mRNA expression increased (Fig. 7B). These results indicated that purified recombinant mouse prolactin retained biological activity. The expressed and purified recombinant mouse prolactin in *E. coli* showed biological activity using *in vitro* 3T3-L1 cell test even though the posttranslational modification was not carried out in *E. coli*. Also, commercial recombinant prolactin (*e.g.*, Assay Genie, Dublin, IE) is also produced in *E. coli*, that meant that the PTMs of mPRL does not necessarily its activity. Moreover, the soluble expression level reached 19.88%, which is relatively high for a eukaryotic protein expressed in *E. coli*, indicating that the absence of PTMs did not hinder proper folding or functional expression in this system [47]. Minor amounts of residual TEV protease may have been present in the final preparation; however, no adverse effects on prolactin activity were observed. Taken together, these findings indirectly support that the recombinant protein acquired a correct and functional three-dimensional structure, sufficient to maintain its native like activity *in vitro*.

Compared to conventional purification methods, this protocol offers several distinct advantages. Previous studies have reported the expression of mouse prolactin as inclusion bodies, requiring denaturation and refolding to obtain functional protein [31], or used complex multi-step purification methods such as RP-HPLC and HPSEC [32]. In contrast, our study achieved soluble expression and established a simple two-step purification method using single MBP affinity chromatography. This simplified purification method reduced the step number of process, eliminated the need for refolding or high-end equipment, and provided sufficient purity and biological activity, highlighting the practical advantages and methodological novelty of the current approach. These advantages are further underlined when compared to the widely used His-tag affinity purification method. The His-tag affinity purification method is widely used due to its simplicity and compatibility with immobilized metal affinity chromatography (IMAC). However, when expressing eukaryotic proteins in *E. coli*, it often results in the formation of inclusion bodies, necessitating additional denaturation and refolding steps to recover biological activity [28, 30]. In some cases, during the his-tag chromatography purification nonspecific binding occurs because of immanent histidine residues in non-target protein, resulting in the binding of the non-target protein to the column [48]. In addition, fused his-tags may affect the activity of the protein by altering its tertiary structure. In contrast, the MBP fusion tag used in this study significantly enhanced protein solubility and folding efficiency, thereby minimizing aggregation and improving the functional yield. A TEV protease cleavage site was inserted between MBP and the prolactin sequence, allowing precise removal of the fusion tag and enabling the recovery of functionally active prolactin without any residual tag. In addition, MBP exhibits high binding specificity for amylose resin and functions as a versatile solubility enhancer across a broad range of target proteins. Although MBP affinity columns are relatively more expensive, their high specificity and reusability contribute to lower long-term purification costs [28-30]. These advantages make the MBP system particularly suitable for the production of complex eukaryotic proteins such as prolactin. Similar systems using fusion tags have demonstrated their versatility across various protein purification needs, further supporting the scalability of this method for diverse applications [39, 49].

## Conclusion

This study focused on the functional validation of biologically active recombinant mouse prolactin. Recombinant mouse prolactin, purified through a simplified strategy using MBP fusion and TEV protease cleavage, exhibited biological activity in cell-based assays utilizing 3T3-L1 cells, indirectly confirming its correct three-dimensional conformation. Thus, the method described herein provides an effective and practical approach for producing functionally active recombinant mouse prolactin suitable for various biological research applications.

## Figures and Tables

**Table 1 T1:** Primer sequences used in this study.

Gene	Direction	Primer sequence (5'–3')
C/EBPβ	Sense	TGGACAAGCTGAGCGACGAG
	Anti-sense	TGTGCTGCGTCTCCAGGTTG
PPARγ	Sense	TTTGACTTTGAGAAATACCC
	Anti-sense	TGGATGAAATTCTCTCCAC
FAS	Sense	TGGGTTCTAGCCAGCAGAGT
	Anti-sense	ACCACCAGAGACCGTTATGC
GAPDH	Sense	AACTTTGGCATTGTGGAAGG
	Anti-sense	ACACATTGGGGGTAGGAACA

**Table 2:** Purification table of recombinant mouse prolactin.

Step	Volume (ml)/(pellet (g))	Total protein (mg)	Target protein (mg)	Purity on SDS-PAGE gel (%)	Step yield (%)	Overall yield (%)
Cell Culture	500.00/(2.45 g)	-	-	-	-	-
Cell Resuspension	61.25	-	-	-	-	-
Supernatant after Cell Lysis	50.00	207.84	-	-	-	-
First MBP Chromatography	59.10	9.60	2.33	81.44	-	100/100
Dialysis and Concentration	8.00	9.60	2.18	76.46	93.56	93.56
TEV Cleavage	10.50	9.45	2.16	22.85	99.08	92.70
Second MBP Chromatography	21.00	0.57	0.57	99.67	26.39	24.46
Dialysis and Concentration	5.25	0.57	0.57	99.66	100.00	24.46
